# A mathematical theory of redox biology

**DOI:** 10.1098/rsos.260547

**Published:** 2026-10-07

**Authors:** James N. Cobley, Michalis G. Nikolaidis

**Affiliations:** ^1^ University of Dundee, Dundee, UK; ^2^ Aristotle University of Thessaloniki, Thessaloniki, Makedonia Thraki, Greece

**Keywords:** redox, curvature, oxidative stress

## Abstract

Redox biology is commonly described through functional labels, such as oxidant, reductant, antioxidant, signalling mediator or damage mediator. However, these labels do not define invariant molecular properties. A molecule may realize different functions depending on reaction partner, local coupling, spatial domain and prior trajectory. Function is therefore not primitive but emergent. Here, we develop a mathematical theory of redox biology from first principles. At the mesoscopic level, chemically defined molecular states are treated as objects and reactions as transformations, yielding an admissible biochemical state space naturally represented as a hypergraph. Function is then defined as a derived relational quantity induced by realized flux over this structural network. As realized flux is temporally mutable, function is necessarily dynamic. By introducing locality, the realized redox state is mathematically represented as an informational occupancy field over space and time. This field is bounded, admits geometric deformation and provides a formal basis for memory, attractors and coarse-grained scalar descriptions like oxidative stress. Measurement and manipulation are treated as coupled informational interfaces to this field, bounding causal inference. The theory yields falsifiable predictions, including flux-dependent function at fixed concentration and history-dependent responses under matched present inputs.

## 1. Introduction

In redox biology, the function of molecules is commonly described by labels, such as oxidant, reductant, antioxidant, mediator of redox signalling or mediator of oxidative damage [1–3]. However, these labels do not identify invariant properties of the molecule. The superoxide anion, for example, can act as either a reductant or an oxidant depending on the reaction partner [4], so neither role can be assigned as an invariant. The problem is widespread. Consider enzymes. Manganese superoxide dismutase (MnSOD) cannot be assigned an invariant antioxidant function [5]. Mis-metalled MnSOD can produce the hydroxyl radical [6], while the active enzyme generates molecular dioxygen and hydrogen peroxide (H_2_O_2_) and therefore depends on other molecules to complete what is described as antioxidant action [7]. Antioxidant is not, therefore, an invariant property of MnSOD. The same logic applies to labels, such as mediator of redox signalling. Even the hydroxyl radical, typically conceived as purely mediating oxidative damage, can mediate redox signalling [8]. These experimentally confirmed examples force a general conclusion: unless a function is universal, it cannot be assigned as an invariant property of a molecule.

As it cannot be assigned *a priori* as an invariant property of a molecule, functions, such as *acts as an antioxidant*, are emergent, context-dependent properties. Ontologically, function is thereby relocated from the molecule considered in isolation to its relational coupling within a biochemical network. As a result, the aim of experiment shifts from uncovering the true role of a molecule, such as the superoxide anion, to identifying the specific contextual conditions under which behaviour emerges, such as *acts as reductant*. Theoretically, this exposes a limitation of the prevailing oxidative stress paradigm [9–15], which does not formalize redox biology on a dynamic, context-dependent basis and therefore has limited predictive power. Oxidative eustress, for example, is commonly treated as though a given level of H_2_O_2_ maps to a defined functional outcome. That mapping cannot be generically injective. The same H_2_O_2_ level may produce a different outcome depending on the context, such as prior exposure to the same.

Here, we present a mathematical theory of redox biology that formalizes function as a dynamic derivative of a first-principled primitive: a structured network of molecular states connected by reactions. Network flux, by encoding the biologically realized chemistry of this network as redox state, dynamically determines function. We conceive the redox state of the network geometrically as a spatio-temporally differentiating field. This redox field is informational: it encodes local redox state mathematically, formalizing occupancy metric: what exists in location x-y-z and time *t*_1_. This informational redox field allows biochemical memory to be formalized through curvature as a mathematical device, not as a physical force. This conceptually uncouples function from concentration. A molecular state present only transiently or at near-zero copy number, such as the hydroxyl radical, may nevertheless imprint the network by changing its redox state—an experimental observable, codified conceptually as geometrically deforming the field on the underlying abstract state space.

This framework generates experimentally testable predictions, including forms of redox hysteresis, and has direct practical implications for experiment. Measurement becomes the observation of a structured projection of the underlying redox field, while manipulation necessarily alters that same field. Causal inference in redox biology is therefore constrained by the fact that observation and intervention are coupled. Because redox trajectories may be path-dependent, the state that initiated a given trajectory may no longer exist at the time of measurement or manipulation. This provides an alternative conceptual basis for why redox causality is often difficult to assign in ageing and disease.

## 2. Results

### 2.1. Overview

In what follows, we construct a first-principled mathematical basis for redox biology organized by structure, function and dynamics. The formal development proceeds from redox objects and their admissible transformations to an informational redox field that encodes occupancy over molecular state space. On this basis, measurement and manipulation can be formalized as distinct but coupled operations on the same underlying redox field. This, in turn, yields experimentally testable predictions, including path-dependent behaviours and forms of hysteresis.

### 2.2. Structure

**Theorem 1**. *At the mesoscopic level of description (table 1), a chemically defined molecular state is an object, a reaction is a transformation, and any transformation that changes one or more invariants maps that object to a distinct object (supplementary material, S1)*.

**Table 1.** Core structural definitions.

term	definition	example
**mesoscopic level of description**	A single-molecule level of description positioned between microscopic and macroscopic descriptions. At this level, a molecule is treated as a chemically defined state with stable identifying descriptors, while unresolved microscopic variation is coarse-grained. This usage is distinct from the condensed-matter physics sense of *mesoscopic* and is introduced here so that object identity can be defined by invariants.	The superoxide anion treated as a chemically defined state with fixed composition, charge and electronic class, rather than as a fluctuating ensemble of underlying quantum microstates or as part of a bulk concentration measurement.
**object**	A chemically defined molecular state specified at the mesoscopic level. An object is identified by a set of invariants sufficient to distinguish it from other admissible molecular states.	Ground-state molecular dioxygen, superoxide anion, hydroperoxyl radical or a defined cysteine proteoform.
**invariant**	A descriptor that contributes to the identity of a mesoscopic object and must be preserved for that object to remain the same object. If one or more invariants change, the resulting state is a distinct object.	For the superoxide anion, invariants include elemental composition (two oxygen atoms), charge state (−1) and electronic configuration (one unpaired electron).
**transformation**	A physically admissible reaction that maps one mesoscopic object to another. A transformation changes one or more invariants and therefore connects distinct objects.	Univalent reduction of molecular dioxygen to the superoxide anion; protonation of superoxide to the hydroperoxyl radical.
**admissible transformation**	A transformation allowed by the chosen chemical representation and consistent with physical law, including relevant conservation laws and permitted state changes.	Electron transfer, proton transfer, excitation, recombination or covalent modification consistent with quantum chemistry and thermodynamics.
**state space**	The set of all admissible mesoscopic objects, together with all admissible transformations connecting them. It represents what may occur in the biochemical system, not what is necessarily realized in a given context.	The admissible oxygen-centred redox state space containing dioxygen, superoxide, hydroperoxyl radical, H_2_O_2_, hydroxyl radical and the reactions connecting them.
**hypergraph**	A structural representation of the admissible state space in which nodes correspond to mesoscopic objects and hyperedges correspond to transformations. A hypergraph is used because reactions may involve multiple inputs and outputs simultaneously.	A redox reaction involving dioxygen, an electron donor and a catalyst can be represented as a hyperedge connecting all participating molecular states.
**morphisms**	The algebraic representation of admissible transformations in the categorical formulation of the theory. Morphisms connect objects and may compose sequentially or in parallel.	A morphism (f:A→B) representing the transformation of molecular dioxygen into superoxide.
**sequential composition**	The chaining of admissible transformations so that the output of one transformation serves as the input to the next.	Dioxygen–superoxide–hydroperoxyl radical.
**parallel composition**	The simultaneous treatment of independent admissible transformations within the same formal system.	One branch in which dioxygen is photoexcited while, independently, another dioxygen molecule is reduced to superoxide.

**Corollary 1**. *A biochemical system therefore admits a structural representation as a state space of chemically defined objects connected by admissible transformations. Because transformations may involve multiple inputs and outputs, this state space is naturally represented as a hypergraph, in which nodes correspond to objects and hyperedges to reactions. This structural representation also admits an algebraic formulation as a symmetric monoidal category, with molecular states as objects and reactions as morphisms (supplementary material, S2). In simple terms, this means that biochemical transformations may be treated within a formal system that accommodates both sequential composition (reaction chains) and simultaneous interaction (parallel reactions).*

Modules.
This hypergraph structure provides a formal scaffold for organizing redox biochemistry. Within this scaffold, objects and transformations can be grouped into three interconnected modules:

1. **The core redox module (CRM):** objects commonly catalogued as core redox molecules, such as reactive oxygen species [16].
2. **The modifying redox module (MRM):** objects that modify molecular states within the CRM, such as superoxide dismutase (SOD) isoforms [17].
3. **The redoxome module (redoxome):** all the other objects that interface through transformations with the CRM and MRM, such as cysteine proteoforms [18–20] (table 1).

**Example.** Consider the superoxide anion (figure 1). The superoxide anion corresponds to a specific molecular state defined by invariant descriptors, including elemental composition (two oxygen atoms), charge (one negative charge) and electronic configuration (one unpaired electron) [21]. Transformations change one or more of these invariants and therefore connect distinct molecular states. For example, protonation of the superoxide anion to the hydroperoxyl radical changes the charge state, thereby transforming one object into another.

**Figure 1.**
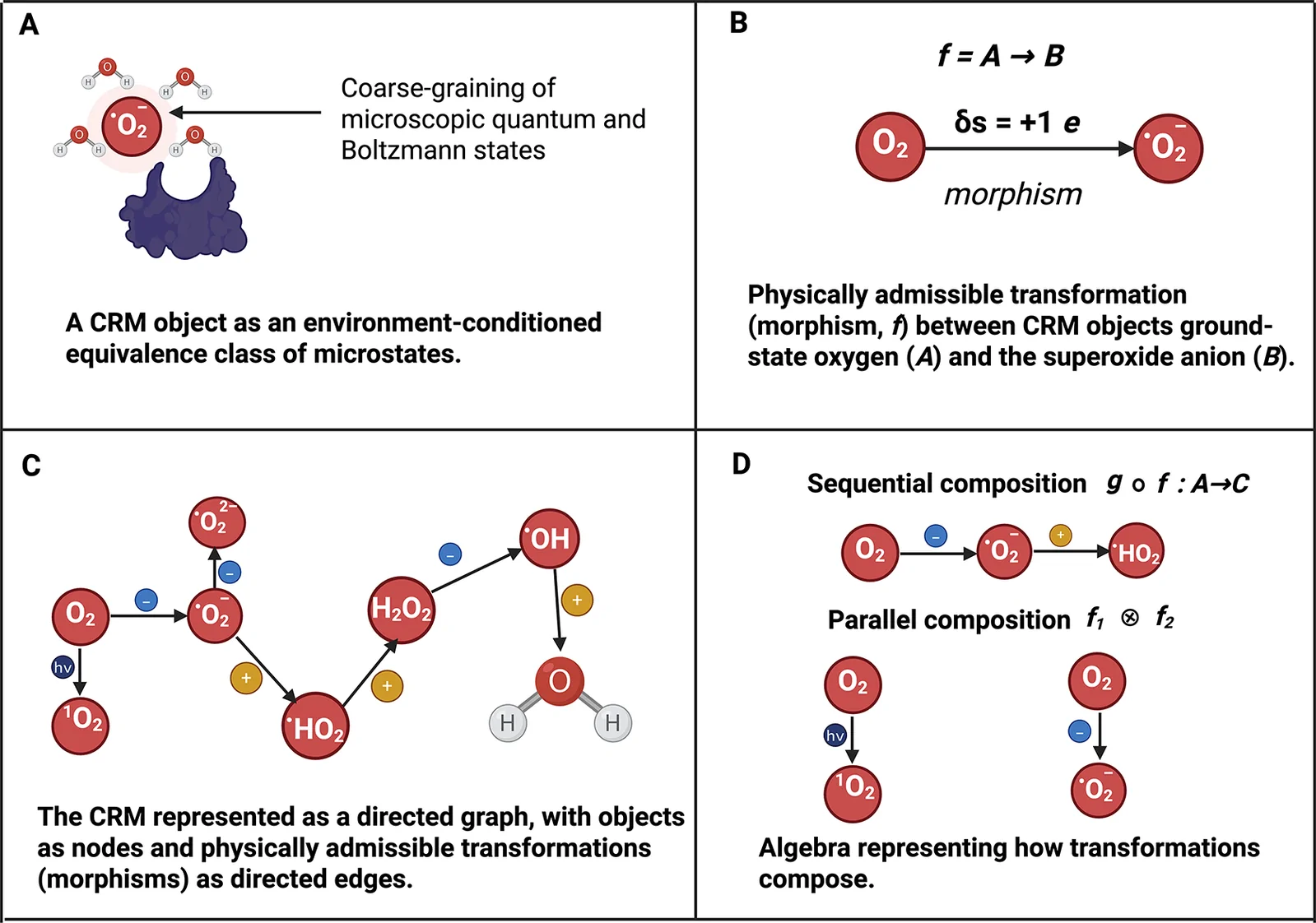
Structural representation of redox biochemistry at the mesoscopic level. (A) A CRM object is defined as an environment-conditioned mesoscopic equivalence class of underlying microscopic quantum and Boltzmann states. (B) A physically admissible transformation is represented as a morphism f:A→B between chemically defined molecular states; here, univalent reduction maps ground-state molecular dioxygen (A) to the superoxide anion (B) by a change in state descriptor (δ*s*  =   + 1*e*). (C) The CRM can be represented structurally as a directed graph in which objects are nodes and physically admissible transformations are directed edges. Illustrated examples include electron transfer, protonation, excitation and downstream reactive transformations linking oxygen-centred species. (D) The same structure admits an algebraic formulation in which transformations compose sequentially (*g*○*f*) or in parallel (*f*_1_⊗*f*_2_), capturing reaction chains and simultaneous interactions within a common formal system. Together, these panels illustrate the structural layer of the theory: molecular states are treated as objects, reactions as transformations and admissible redox biochemistry as a composed state space.

The transformations in which the superoxide anion participates are represented as hyperedges connecting it to other objects in the hypergraph. For example, a hyperedge connects ground-state molecular dioxygen to the superoxide anion through a univalent reduction reaction [22], the transfer of a single electron, thereby transforming one CRM object into another. The electron must be supplied by another object, such as complex I, which belongs to the MRM. Transformations involving the superoxide anion may also connect to the redoxome. For example, through reactions with proteins, such as an aconitase proteoform [23–25].

The hypergraph encodes the admissible superoxide anion-centred state space independently of which transformations are realized in a given context. Functional labels, such as oxidant or reductant, do not enter the structural identity of the object, unless they are an invariant.

### 2.3. Function

**Theorem 2**. *At the macroscopic level of description, function is a derived relational quantity induced by realized transformations within the biochemical network. Let *G*  =  (*V*, *E*) denote the admissible structural network, where *V* is the set of objects and *E* the set of admissible transformations, and let $w:E\to R_{\geq 0}$ assign non-negative realized flux weights to those transformations. The realized function of an object vϵV is then defined by its contribution to the flux-induced change in the network redox state*,

$$\begin{array}{r} f\left(v;w\right):\;=\;\Delta R_{v}\left[w\right]. \end{array}$$

Functional labels are therefore secondary annotations of this derived quantity. Function arises through realized flux over the structural primitive (supplementary material, S3).

In a system-specific implementation, the realized weight *w_t_*(*e*) associated with transformation *e* may be identified with its local reaction flux. This flux is determined by local molecular-state occupancy, kinetic parameters, enzyme activity, transport and boundary conditions [26]. Transport may be represented by ordinary diffusion or, where experimentally justified, by anomalous or memory-dependent transport [27].

**Corollary 2**. *Structural identity determines the admissible functional possibilities of an object, but realized function is determined only by the flux-weighted pattern of transformations realized in context*.

**Corollary 3**. *Function is irreducible to concentration alone. As realized function is defined by flux-induced change in network redox state, transient objects may carry substantial function. Similarly, an abundant object may carry none depending on the context*.

**Corollary 4**. *The projection from realized function to verbal functional label is generally non-injective. Distinct realized flux distributions and distinct network redox states may therefore be assigned the same coarse-grained label, such as acted as an antioxidant*.

**Example**. H_2_O_2_ may react with a catalytic cysteine thiolate in peroxiredoxin-2 within the MRM or with a cysteine residue in a redoxome protein, such as phosphatase and tensin homologue (PTEN) [28–30] (figure 2). These distinct transformations generate distinct cysteine proteoforms.

**Figure 2.**
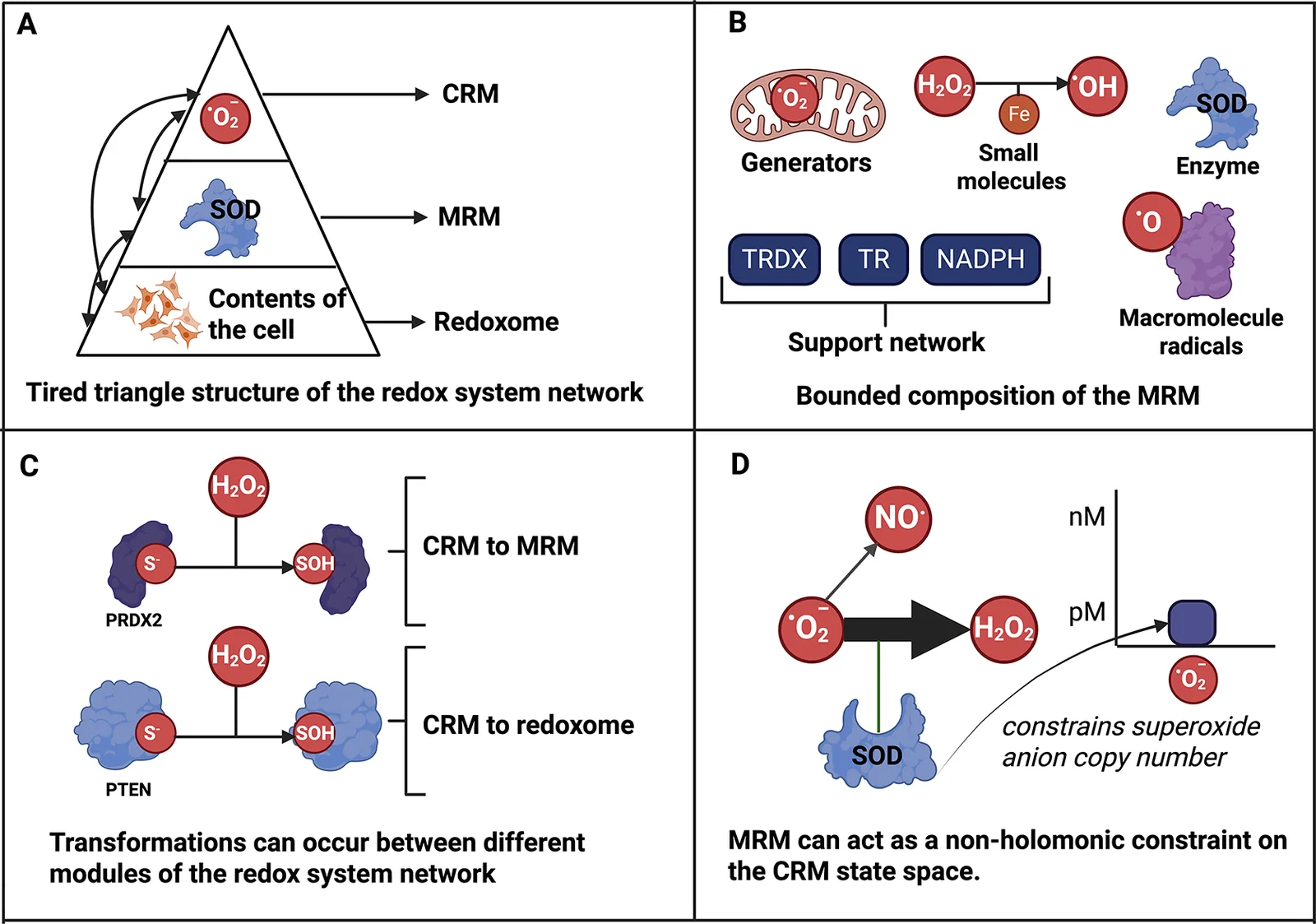
Emergence of redox function through modular constraint of flux. (A) Tiered organization of the redox system network. The CRM defines the admissible space of small-molecule redox states and transformations. The MRM comprises enzymes and cofactors that constrain or mediate CRM transformations. The redoxome comprises the remaining cellular contents capable of interacting with CRM objects. Function emerges from constrained interactions across these layers. (B) Bounded composition of the MRM. The MRM consists of a finite set of generators, enzymes, small-molecule mediators and support networks that interface directly with CRM transformations. This bounded composition channels flux through the CRM without altering the underlying admissible state space. (C) Transformations across network modules. The same CRM object may participate in transformations mediated by the MRM (top) or in direct interactions with redoxome components (bottom). These distinct transformation contexts yield distinct biological consequences despite originating from the same admissible CRM state. (D) Constraint of realized redox trajectories by the MRM. Enzymatic activity, exemplified here by SOD, does not remove states from the CRM but biases realized flux towards specific transformations. The MRM therefore constrains accessible trajectories through the CRM state space without redefining that space structurally.

Realized function depends on flux allocation over this admissible structure. SOD isoforms bias realized flux towards morphisms converting the superoxide anion to H_2_O_2_ and ground-state dioxygen. Alternative admissible transformations, such as reaction with nitric oxide to form peroxynitrite [31], therefore carry reduced realized flux under these conditions. The admissible state space is unchanged, but the realized trajectory through it is constrained. The constraint therefore operates at the level of flux rather than object identity.

### 2.4. Dynamics

**Theorem 3**. *Because realized flux on the admissible structural network is temporally mutable, realized function is necessarily time-dependent. Let*

$$\begin{array}{r} w_{t}:E\to R_{\geq 0} \end{array}$$

denote the realized flux distribution at time *t*. Since realized function is defined by

$$\begin{array}{r} f_{t}\left(v\right):\;=\;\Delta R_{v}\left[w_{t}\right], \end{array}$$

function evolves whenever the realized flux distribution evolves (supplementary material, S4).

**Corollary 5**. *A biochemical system therefore realizes a trajectory through redox state space. Dynamics define changes in the realized flux pattern over that layer through time*.

**Corollary 6**. *Path-dependence arises whenever realized function at time t depends not only on the present realized flux distribution w_t_*, *but also on the prior history {w_s_}_s < *t*_*. *In such cases, identical present structural conditions need not imply identical realized function*.

**Corollary 7**. *Hysteresis arises when the realized redox state or realized function under a given present condition depends on the trajectory by which that condition was reached. Accordingly, identical present control conditions may correspond to distinct realized functional states*.

When kinetic information is incomplete, structural, stoichiometric and conservation constraints may define a feasible set of flux configurations rather than a unique trajectory. Flux balance or related constraint-based approaches therefore provide one possible implementation, particularly near steady state. They are not, however, uniquely privileged by the theory. Spatially distributed, transient and history-dependent behaviour instead requires kinetic, reaction–diffusion, stochastic or memory-augmented formulations.

**Example**. Consider H_2_O_2_ interacting with peroxiredoxins (PRDX), the thioredoxin system (TRX) and cysteine-containing redoxome targets (figure 3). H_2_O_2_ participates in multiple admissible transformations, including enzymatic reduction via PRDX, regeneration through the TRX system and oxidation of redoxome targets [32–34].

**Figure 3.**
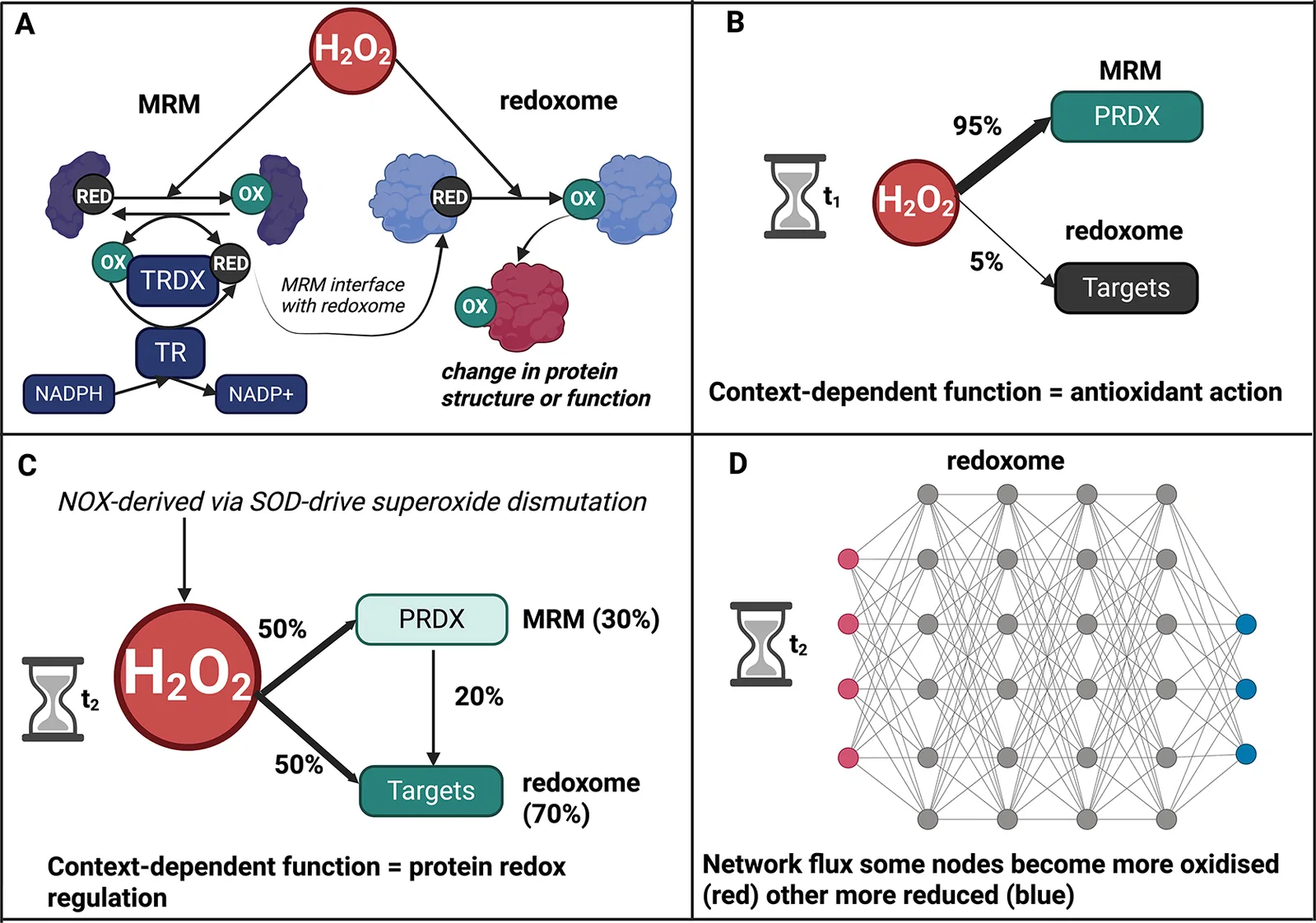
Redox dynamics arise from time-dependent redistribution of realized flux over a fixed admissible network. (A) A H_2_O_2_-centred subsystem spanning the MRM and the redoxome. H_2_O_2_ participates in multiple admissible transformations, including enzymatic reduction via PRDX, regeneration through the TRX system and oxidation of redoxome protein targets. (B) At time *t*_1_, realized flux from H_2_O_2_ is predominantly allocated to PRDX-mediated transformations within the MRM, with a smaller fraction coupled to redoxome targets. Under these conditions, the realized function is dominated by antioxidant action. (C) Following a perturbation that elevates H_2_O_2_ production, for example, through NOX activity with SOD-driven dismutation, the admissible network remains unchanged but the realized flux distribution is reweighted at time *t*_2_. A greater fraction of flux is thereby allocated to redoxome-target transformations, shifting the realized function towards protein redox regulation. (D) Reweighting of realized flux alters the redox state of downstream network nodes without introducing new molecular states or new transformations. Dynamics therefore arise from temporal redistribution of flux over an invariant structural primitive, and function emerges from this changing realization rather than from intrinsic molecular identity.

At time *t*_0_, suppose that 95% of H_2_O_2_-derived flux is allocated to PRDX-mediated transformations and 5% to oxidation of redoxome targets. This realized weighting defines the instantaneous functional state of the system.

Following a perturbation that increases H_2_O_2_ production, the admissible structure remains unchanged, but the realized flux distribution is redistributed. At time *t*_1_, a larger fraction of flux is allocated to redoxome-target transformations. The realized system state is therefore altered solely through reweighting of existing morphisms.

As no new molecular states or transformations are introduced, the change arises entirely from time-dependent redistribution of realized flux within a fixed structure.

### 2.5. Geometry

**Theorem 4**. *The realized redox state admits a granular local representation as an occupancy field over space and time. Let*

$$\begin{array}{r} \rho \left(x,y,z,t\right) \end{array}$$

*denote the realized occupancy of admissible redox state at spatial position (*x*, *y*, *z*) and time *t* (supplementary material, S5). The biochemical system may therefore be represented by an informational redox field that specifies what is realized locally across the domain. When multiple local state occupancies are tracked simultaneously, this field may be treated as vector-valued*.

*This redox field is not a new physical field and does not imply a new biochemical mechanism. It is a mathematical device for introducing locality into the structural and dynamical formalism by encoding the local realized redox state*.

*The geometric redox field introduced here is related to, but distinct from, what is commonly termed a redox landscape. The field represents the realized redox state distributed across physical space and time, whereas the landscape describes the organization of possible states, transitions, basins and attractors in the underlying state or configuration space. The temporal evolution of the realized field may therefore be represented as a trajectory through the associated redox landscape*.

**Corollary 8**. *Local realized occupancy is bounded by construction*,

$$\begin{array}{r} 0\leq \rho \left(x,y,z,t\right)\leq \rho_{max}\left(x,y,z,t\right). \end{array}$$

The informational redox field therefore inhabits a bounded state space rather than an unbounded linear one. This boundedness naturally supports piecewise-linear, including tropical, descriptions of dynamics.

**Corollary 9**. *Geometric structure may then be imposed on this occupancy field. Curvature may be introduced as a mathematical metric for describing local deformation of realized redox state, providing a formal basis for modelling persistence, memory, attractors and related path-dependent behaviour*.

**Corollary 10**. *Any scalar descriptor of redox biology is necessarily a low-dimensional projection of an underlying high-dimensional realized redox state. Let*

$$\begin{array}{r} X \end{array}$$

denote the full realized redox configuration, for example, including local occupancy, realized flux and temporal state. A scalar descriptor is then a projection

$$\begin{array}{r} \pi \left(X\right)\epsilon R, \end{array}$$

or more generally a low-dimensional summary

$$\begin{array}{r} \pi \left(X\right)\epsilon R^{k},k\ll dim\left(X\right). \end{array}$$

Terms such as oxidative stress or coarse functional labels therefore compress a spatially distributed, dynamically evolving and locally heterogeneous system into a reduced description [35,36].

**Corollary 11**. *Such projections are generally non-injective. Distinct realized redox configurations may therefore yield the same scalar descriptor*,

$$\begin{array}{r} X_{1}\neq X_{2}but\;\pi \left(X_{1}\right)\;=\;\pi \left(X_{2}\right). \end{array}$$

Accordingly, identical scalar descriptions need not imply identical underlying redox states. Distinct nanodomain configurations, flux histories or local occupancy patterns may collapse to the same term, such as oxidative stress or *acted as an antioxidant*.

**Corollary 12**. *There is no uniquely privileged redox state or reference frame [37]. Let *X* denote a realized redox configuration and *X*_ref_ a chosen reference state, so that interpretation is often expressed relative to*

$$\begin{array}{r} \Delta X\;=\;X-X_{ref}. \end{array}$$

Because realized redox biology is relational, flux-dependent, spatially heterogeneous and path-dependent, no single *X*_ref_ can serve as a universal invariant baseline across contexts. Any reference state is therefore a modelling or experimental convenience rather than a privileged ontological origin.

For a specified redox couple, a reduction potential may be derived when the relevant thermodynamic assumptions are satisfied. Such a value is nevertheless a derived, couple-specific descriptor and should not automatically be interpreted as the controlling variable of a biological redox process. More generally, a spatially heterogeneous, open and kinetically controlled biological system does not generally admit one unique overall redox potential. Any bulk potential-like readout is therefore a couple-, domain- and measurement-specific projection of the underlying molecular-state occupancies and fluxes, and distinct underlying states may produce the same measured value.

**Example** **.** Consider H_2_O_2_ distributed across multiple spatial domains. Although the admissible transformations of H_2_O_2_ are fixed, local flux distributions may differ between domains due to variation in enzyme abundance and transport conditions (figure 4). At a given time, distinct spatial regions may exhibit different weighted configurations despite sharing identical admissible structure. Temporal evolution at each location is governed by the same dynamical law but subject to local boundary conditions.

**Figure 4.**
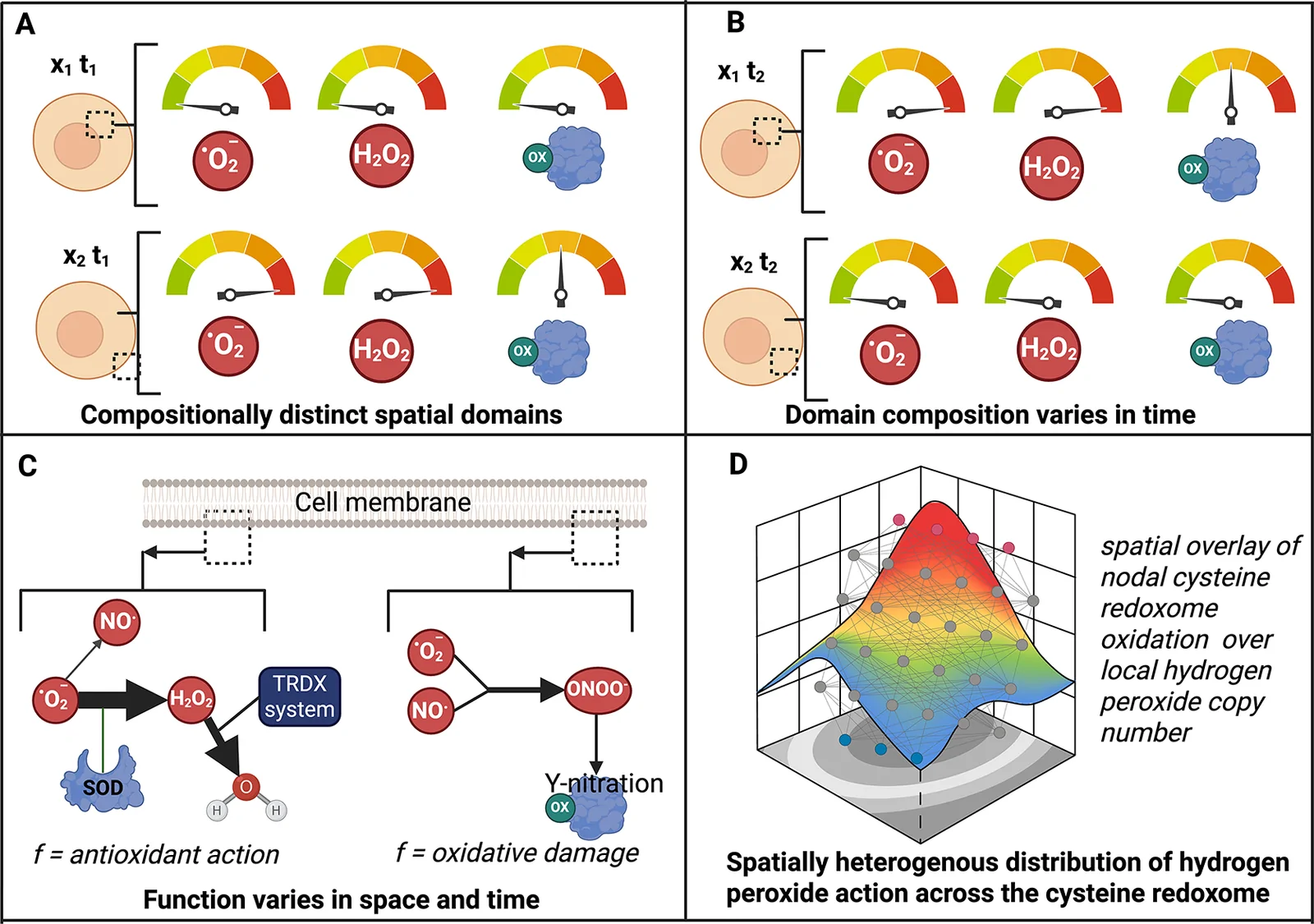
Geometric redox field. (A) Compositional heterogeneity across space. Distinct spatial domains (*x*_1_, *x*_2_) carry locally instantiated redox systems with identical algebraic structure but different flux distributions across CRM, MRM and redoxome transformations at the same time point (*t*_1_). Local redox state is determined by the relative weighting of admissible transformations rather than by molecular identity alone. (B) Temporal evolution of local redox composition. At a fixed spatial location, flux distributions evolve over time (*t*_1_ → *t*_2_), deforming the local redox state without altering the underlying transformation structure. This illustrates that redox state and function are inherently time-dependent. (C) Spatially localized emergence of function. The same CRM object (e.g. H_2_O_2_) participates in different dominant transformation pathways depending on spatial embedding and local constraints. In one domain, flux is channelled through the MRM to yield antioxidant metabolism; in another, alternate morphisms dominate, leading to oxidative damage. Some morphisms involving free radical objects like the nitrogen dioxide radical that lead to tyrosine nitration are omitted for visual clarity. Function therefore varies across space. (D) Field-level view of spatial redox organization. Local redox states form a spatially heterogeneous field, illustrated here as the overlay between nodal cysteine redoxome oxidation and local H_2_O_2_ copy number. Biological outcomes emerge from the coupled evolution of flux distributions across space and time.

### 2.6. Information—measurement, manipulation and causality

Definition 1
**(*Redox information*).** *Redox information is the experimentally accessible projection of the realized redox field within a specified spatial and temporal domain. If *X* denotes the full realized redox configuration and *M* a measurement operator, then the measured datum is*

$$\begin{array}{r} I\;=\;M\left(X\right). \end{array}$$

Because *M* samples only a restricted subset of the field, redox information is necessarily partial.

Definition 2
**(*Redox measurement*).** *A measurement is an operation that maps the realized redox field into an observable domain under finite spatial, temporal and molecular resolution [38]. Measurement therefore does not access the full realized field, but only a structured projection of it. In general*,

$$\begin{array}{r} M:X\to Y, \end{array}$$

where *Y* is a lower-dimensional observable space [38]. Because measurement is physically implemented, it may alter the system before the readout is obtained. Let *U_M_* denote the perturbation induced by the measurement procedure and *M_R_* the subsequent readout operation. The measured datum is therefore

$$\begin{array}{r} I_{M}=M_{R}\;\left(U_{M}\left(X\right)\right). \end{array}$$

A non-perturbative measurement is the limiting case in which *U_M_*(*X*) = *X*.

Definition 3
**(*Redox manipulation*).** *A manipulation is a deliberate perturbation of the realized redox field within a specified domain. If *U* denotes such an intervention, then manipulation acts by altering realized flux, state occupancy or local coupling structure*,

$$\begin{array}{r} X\mapsto U\left(X\right). \end{array}$$

Manipulation does not introduce a new ontology. It redistributes the realized deployment of admissible transformations, which are evolving in time per dynamics.

Definition 4
**(*Redox causation*).** *Causal inference arises from the relation between manipulation and measurement. If *U* is a manipulation and *M* a measurement, then causal interpretation is based on the comparison under controlled boundary conditions. Causation is inferred from the change in projected observables induced by a defined perturbation*,

$$\begin{array}{r} M\left(U\left(X\right)\right)-M\left(X\right). \end{array}$$

**Corollary 13**. *Measurement and manipulation are dual informational interfaces with the realized redox field. Measurement determines what can be seen; manipulation determines what can be changed. Causal inference is therefore bounded by the intersection of these domains*.

**Corollary 14**. *Because M is a projection, causal inference is necessarily conditional and generally non-unique. Distinct underlying redox configurations or trajectories may yield the same measured outcome*,

$$\begin{array}{r} X_{1}\neq X_{2}\;but\;M\left(X_{1}\right)\;=\;M\left(X_{2}\right). \end{array}$$

Accordingly, experimental readouts do not recover the full realized redox field, but only a method-defined and potentially perturbative projection of it.

When the composite measurement map *M_R_*○*U_M_* is non-injective, the precise pre-measurement state cannot be uniquely recovered from the measured datum alone. It may nevertheless be estimated or bounded through calibration of measurement back-action, variation of measurement strength, complementary methods or mechanistic modelling. Calcium indicators that buffer intracellular calcium and patch-clamp procedures that alter membrane behaviour provide analogous examples.

### 2.7. Falsifiable predictions

The theory becomes falsifiable at the level of realized flux, temporal evolution and trajectory dependence. The following predictions follow directly from the structure–function–dynamics formalism.

Prediction 1.
**Function depends on realized flux, not concentration alone.** Let a CRM object be present at the same concentration in two systems with the same admissible structural layer. If the local coupling environment differs, for example, through differences in enzyme abundance, cofactor availability, transport or network connectivity, then the realized flux distribution will differ. The theory therefore predicts that identical concentration need not yield identical realized function or identical downstream biological observables.

Prediction 2.
**Prior trajectory alters subsequent response.** If two systems are brought to the same present input condition through different prior redox histories, then their subsequent realized redox states need not be identical. The theory therefore predicts history-dependent response, even when present molecular composition and applied perturbation are matched.

Prediction 3.
**Hysteresis and order sensitivity are generic consequences of memory.** If redox memory is non-zero, then restoration of the same external condition does not, in general, imply return to the same realized redox state. Similarly, sequential perturbations need not commute: applying perturbation *A* followed by *B* need not produce the same outcome as applying *B* followed by *A*. The theory therefore predicts hysteresis and order-of-perturbation effects as structural consequences of flux evolution on a memory-bearing field.

A direct experimental test is a repeated H_2_O_2_ challenge in matched cultures with and without prior exposure. The theory predicts that the second response will depend on prior trajectory, even if the present challenge is identical. More generally, systematic failure of the three predictions above under controlled conditions would falsify the curvature-extended dynamical framework.

## 3. Discussion

The central motivation for this work is that a conceptual basis of redox biology predicated on invariant functional labels cannot serve as a first-principles foundation for the field. Labels such as oxidant, reductant, antioxidant, signalling mediator or damage mediator are operationally useful, but they do not identify invariant molecular properties. Function is mutable. It depends on reaction partner, coupling structure, local environment, spatial domain and prior trajectory. A molecule may therefore realize different functions in different contexts without any change in its underlying molecular identity. The current working paradigm is consequently not generically adequate as a foundational description, because it treats emergent behaviours as though they were primitive descriptors.

The present theory begins from different primitives. Defined molecular states are treated as objects and reactions as transformations, yielding a lawful representation of admissible biochemistry independent of whether particular behaviours are realized in a given context. Function then appears only at the level of realized flux. It is not an intrinsic property of the object, but a derived relational quantity induced by the weighted pattern of transformations that change the redox state of the network. In this framework, structure specifies possibility, whereas realized flux specifies behaviour.

Once function is treated as derived rather than primitive, several consequences follow. First, concentration alone cannot determine function, because abundance does not specify how admissible transformations are weighted [39]. Second, dynamics become necessary rather than optional, since realized flux is temporally mutable and function therefore evolves in time. Third, geometry becomes integral to description, because realized redox state is spatially distributed and locally heterogeneous [40,41]. The informational redox field introduced here formalizes this locality without postulating a new physical field or biochemical mechanism. Curvature is then introduced not as a force, but as a mathematical device for describing deformation, persistence and memory in realized redox state.

This shift in primitives also changes the interpretation of experiment. Measurement does not recover the realized redox field in full, but only a structured projection of it under finite spatial, temporal and molecular resolution. Manipulation perturbs that same field by altering realized flux, local occupancy or coupling relationships. Causal inference is therefore neither molecule-centric nor absolute. It is conditional on the relation between perturbation and projection. What can be inferred depends jointly on what can be changed and what can be seen. This provides a formal basis for why redox causality is often difficult to assign uniquely in ageing, disease and adaptation [42]: the states and trajectories that generated an observed phenotype may no longer exist, or may no longer be experimentally accessible, at the point of measurement.

The theory is falsifiable because its central claims are experimentally testable. If function depends on realized flux rather than concentration alone, then identical concentrations of a redox-active object placed in different coupling environments should yield different realized functions and different downstream observables. If dynamics are path-dependent, then systems exposed to identical present inputs but different prior redox histories should not, in general, produce identical subsequent responses. If memory is non-zero, then hysteresis and order sensitivity should arise: restoring identical external conditions need not restore identical realized states, and sequential perturbations need not commute. These predictions follow from the formal structure of the theory itself. A systematic failure of such effects under controlled conditions would count against the framework.

The scope of the theory should also be clear. This is a first-principles mathematical account, not a completed system-specific model. The theory specifies the relevant objects, relations and testable consequences; it does not assign universal numerical values to quantities that are explicitly system-, location-, time- and history-dependent. Numerical prediction of occupancies and fluxes requires a separate system-specific implementation incorporating the relevant kinetics, transport, boundary conditions and prior state. Failure of a particular parametrization or numerical implementation would not, by itself, invalidate the general theory, unless it demonstrated failure of a consequence that follows from the theory's formal structure. It does not claim that every biochemical system must be represented with the same descriptor set, field construction or geometric metric. Those choices remain representation-dependent and may vary with scale, system and experimental question. The informational redox field is not a new physical field, and curvature is not a new biochemical force. Both are formal devices. Their value lies in explanatory and predictive power: they provide a disciplined way to represent locality, bounded occupancy, deformation, memory, attractors and scalar coarse-graining within a single framework. In the same way, the present theory does not abolish conventional redox terms. It reassigns their status. Labels such as oxidant, antioxidant, oxidative stress or eustress remain useful operational summaries, but they can no longer be treated as invariant descriptors or privileged explanatory primitives. Their correct place is downstream of structure, realized flux, dynamics and geometry.

A final consequence of the theory is that scalar redox descriptors must be interpreted with caution. Once realized redox state is understood as spatially distributed, dynamically evolving and locally heterogeneous, any scalar term becomes a low-dimensional projection of a higher-dimensional system. Terms such as oxidative stress therefore compress nanodomain heterogeneity, trajectory dependence and local flux structure into coarse summaries. This does not make them useless. It means they are non-unique. Distinct realized redox configurations may collapse to the same scalar description, and no uniquely privileged redox state or reference frame exists from which all function can be measured invariantly. Any baseline is therefore a modelling or experimental convenience rather than an ontological origin.

## 4. Conclusion

The main claim of this work is that function in redox biology is a realized, relational and context-dependent consequence of admissible transformations unfolding through time and space. From this starting point, we developed a mathematical theory in which chemically defined molecular states are treated as objects, reactions as transformations, function as flux-induced change in network redox state and locality as an informational occupancy field. This reframing replaces static molecular role labels with a first-principles account grounded in structure, realized flux, dynamics, geometry and bounded inference. If correct, it shifts redox biology away from fixed functional attribution and towards the disciplined study of structured possibility, realized trajectory and the evolving biochemical field through which function emerges.

## Supplementary Material



## Data Availability

Data and relevant code for this research work are stored in GitHub: https://github.com/JamesCobley/Redox_Maths and have been archived within the Zenodo repository [43]. Supplementary material is available online [44].
