# Supplementary material for: A mathematical theory of redox biology
Source: R Soc Open Sci. 2026 Oct 7;13(10):260547. doi: 10.1098/rsos.260547 (PMC13641906; doi:10.1098/rsos.260547)
Supplement: Supplementary file 1 [file rsos.260547.zip › rsos.260547/rsos260547_si_001.docx]

**Supplemental Notes**

**Supplementary Note 1.**

**Proposition.**
Let object identity at the mesoscopic level be determined by a specified set of invariants $I(o)$. Let a reaction be a map between molecular states. If a reaction changes one or more invariants, then the image of that reaction is a distinct object.

**Proof sketch.**
By definition, two mesoscopic objects are identical only if they share the same invariant set. If a reaction changes one or more invariants, then the resulting state has an invariant set different from that of the starting state. The product state is therefore not identical to the starting state and must be treated as a distinct object. Since reactions may involve multiple inputs and outputs, the admissible biochemical system is naturally represented as a hypergraph, whose nodes are objects and whose hyperedges are reactions. $\square$

**Supplementary Note 2.**

The admissible redox state space may also be expressed algebraically as a **small symmetric monoidal category** $\mathcal{C}_{\mathrm{adm}}$. In this formulation, objects represent discrete mesoscopic molecular state classes and morphisms represent physically admissible transformations between them. This formalism captures not only which state classes and transformations are allowed, but also how transformations compose sequentially and combine in parallel.

**S2.1 Objects**

Each object $A\in\mathrm{Obj}(\mathcal{C}_{\mathrm{adm}})$ denotes a mesoscopic molecular state class. Such objects are specified by chemically relevant descriptors sufficient to distinguish one admissible state from another at the chosen level of representation. These may include elemental composition, charge state, protonation class, bonding configuration, and electronic class.

**S2.2 Morphisms**

A morphism $f:A\to B$denotes a physically admissible transformation from state class $A$to state class $B$. Morphisms therefore encode reaction classes consistent with the structural resolution adopted in the theory.

**S2.3 Admissibility**

Only transformations consistent with the relevant physical and chemical constraints are included in $\mathcal{C}_{\mathrm{adm}}$. Admissible morphisms respect conservation laws and allowed state transitions at the chosen mesoscopic resolution.

**S2.4 Sequential and parallel composition**

Composition of morphisms,

$$g\circ f:A\to C,$$

represents sequential chaining of admissible transformations. The monoidal product,

$$A\otimes B,$$

represents parallel composition, that is, the joint presence of molecular state classes within a composite local system. Likewise,

$$f_{1}\otimes f_{2}:A_{1}\otimes A_{2}\to B_{1}\otimes B_{2},$$

represents independent concurrent transformations. Symmetry means that parallel composition is insensitive to ordering up to canonical equivalence.

This algebraic formulation is therefore equivalent in purpose to the hypergraph representation used in the main text, but emphasises the compositional structure of admissible biochemistry.

**Supplementary Note 3.**

Let the admissible biochemical structure be represented by a directed network

$$G=(V,E),$$

where $V$ is the set of mesoscopic objects and $E$the set of admissible transformations between them.

To distinguish structural possibility from realised behaviour, let

$$w:E\to\mathbb{R}_{\geq0}$$

be a realised flux weighting on admissible transformations. For each $e\in E$, $w(e)=0$denotes an admissible but unrealised transformation, whereas $w(e)>0$ denotes realised non-negative flux through that transformation.

Let

$$R[w]$$

denote the redox state of the network induced by the realised flux distribution $w$. The precise form of $R[w]$may depend on the chosen representation, but in all cases it is a functional of realised flux over the admissible structural network.

For an object $v\in V$, define its realised function by

$$f(v;w):=\Delta R_{v}[w],$$

where $\Delta R_{v}[w]$ denotes the contribution of flux-carrying transformations involving $v$to the change in network redox state. Function is therefore not assigned from object identity alone, but derived from realised relational behaviour within the network.

This formalism excludes any primitive object-to-label assignment

$$V\to L,$$

where $L$is a set of verbal role labels such as “oxidant,” “reductant,” or “antioxidant.” Such labels are instead treated as coarse-grained projections of the derived functional quantity:

$$\Lambda\mathcal{:F\to}L,\mathcal{l}_{v}=\Lambda(f(v;w)),$$

where $\mathcal{F}$ denotes the space of realised functional quantities and $\mathcal{l}_{v}\in L$ the assigned label.

The consequence is that structural identity defines only admissible functional possibility, whereas realised function depends on the contextual flux distribution. Thus, identical objects may carry different realised functions under different flux patterns, and similar functional labels may arise from distinct underlying network trajectories.

**Proposition 3.1**

If $w(e)=0$ for all transformations incident on object $v$, then no realised function is assigned to $v$ at that level of description.

**Proof sketch**

If no realised flux passes through any admissible transformation involving $v$, then $v$contributes no change to network redox state, so $\Delta R_{v}[w]=0$ in the realised system. Function is therefore not realised, even if structurally admissible. $\square$

**Proposition 3.2**

Realised function is context-dependent: the same object may be assigned different functional labels under different realised flux distributions $w_{1}\neq w_{2}$.

**Proof sketch**

Since $f(v;w)=\Delta R_{v}[w]$, changing the realised flux distribution changes the induced network redox contribution of $v$. The corresponding coarse-grained label $\Lambda(f(v;w))$may therefore also change. $\square$

**Proposition 3.3**

The label projection

$$\Lambda\mathcal{:F\to}L$$

is generally non-injective.

**Proof sketch**

A coarse-grained label discards information about the full realised functional quantity $f(v;w)$. Distinct functional quantities may therefore map to the same label. Since $f(v;w)$is induced by realised flux and network redox state, distinct flux distributions and distinct redox states may likewise yield the same verbal classification. $\square$

**Proposition 3.4**

Concentration alone does not determine realised function.

**Proof sketch**

Realised function depends on $\Delta R_{v}[w]$, which is induced by realised flux through admissible transformations, not abundance alone. Low-abundance states may alter network redox state strongly if their realised transformations are decisive, whereas abundant states may have no realised contribution under the same structural conditions. $\square$

**Supplementary Note 4.**

Let the admissible structural network be

$$G=(V,E),$$

where $V$is the set of mesoscopic objects and $E$the set of admissible transformations. Dynamics enter not through redefinition of this structural primitive, but through time-dependent realisation of flux over it.

**S4.1 Time-dependent realised flux**

Let

$$w_{t}:E\to\mathbb{R}_{\geq0}$$

denote the realised non-negative flux weighting on admissible transformations at time $t$. For each transformation $e\in E$, $w_{t}(e)$specifies the extent to which that admissible transformation is realised at time $t$. The structural layer $G$is fixed at the chosen level of description; dynamics arise from temporal variation in $w_{t}$.

**S4.2 Time-dependent redox state**

Let

$$R_{t}:=R[w_{t}]$$

denote the redox state of the network induced by the realised flux distribution at time $t$. Thus the redox state is treated as a functional of realised flux over the admissible structure.

**S4.3 Time-dependent realised function**

For an object $v\in V$, define realised function at time $t$by

$$f_{t}(v):=\Delta R_{v}[w_{t}],$$

where $\Delta R_{v}[w_{t}]$denotes the contribution of realised flux involving $v$to the change in network redox state at time $t$. Realised function is therefore time-dependent whenever the realised flux weighting is time-dependent.

**S4.4 Realised trajectories**

A realised redox trajectory is a time-ordered family

$$\left\{ w_{t}{\}}_{t\in T} \right,$$

or equivalently the induced family of redox states

$$\left\{ R_{t}{\}}_{t\in T}. \right.$$

Dynamics are therefore changes in realised flux over a fixed admissible structure.

**S4.5 Path dependence**

If realised function at time $t$depends only on the current flux weighting $w_{t}$, then the system is memoryless at that level of description. If realised function depends on the prior history

$$\left\{ w_{s}{\}}_{s<t} \right,$$

then the trajectory is path-dependent.

**S4.6 Hysteresis**

Hysteresis arises when identical present control conditions correspond to different realised functions or redox states because those states depend on prior flux history. In this framework, hysteresis is therefore a property of realised trajectories over the structural primitive rather than of structure alone.

**Supplementary Note 5**

The geometry layer introduces locality into the theory by representing realised redox state as an informational occupancy field over space and time. This construction does not introduce a new physical field or biochemical mechanism. It is a mathematical device for encoding what redox states are locally realised, and with what occupancy, across a spatial domain.

**S5.1 Spatial domain and local occupancy**

Let

$$\Omega\subset\mathbb{R}^{3}$$

denote the spatial domain occupied by the biochemical system, for example a cellular or tissue volume. At each position

$$x=(x,y,z)\in\Omega$$

and time $t$, the realised redox state is represented by a local occupancy field

$$\rho(x,t).$$

More explicitly, if the admissible redox state space is indexed by $i\in\mathcal{S}$, then the local field may be written componentwise as

$$\rho_{i}(x,t),i\in\mathcal{S,}$$

where $\rho_{i}(x,t)$ denotes the realised local occupancy of admissible state $i$ at location $x$ and time $t$. When multiple occupancies are tracked simultaneously, the local redox field is therefore naturally vector-valued:

$$\rho(x,t)=(\rho_{i}(x,t))_{i\in\mathcal{S}}.$$

This field specifies what is locally realised across the domain. It is informational rather than physical a force-carrying field.

**S5.2 Bounded occupancy**

Local occupancy is bounded by construction. For each admissible state $i$,

$$0\leq\rho_{i}(x,t)\leq\rho_{i,\max}(x,t),$$

where $\rho_{i}\left( x,t \right)=0$ corresponds to absence of state $i$ at location $x$ and time $t$, and $\rho_{i,\max N}\left( x,t \right)$denotes maximal local realisation of that state at the chosen level of description.

Thus, the local redox field inhabits a bounded state space rather than an unbounded linear one. This boundedness is a direct consequence of the occupancy construction and not an additional assumption.

**S5.3 Tropical and piecewise-linear interpretation**

Because the occupancy field is bounded, non-negative, and locally saturable, it naturally admits piecewise-linear descriptions. Local redox evolution may be interpreted in tropical terms whenever max–min or related bounded operations better reflect admissible state transitions than unrestricted linear superposition.

The point here is not that the geometry layer is intrinsically tropical in every formulation, but that bounded occupancy makes tropical and piecewise-linear descriptions mathematically natural. This is especially relevant when transitions are constrained by local saturation, exclusion, or competition between admissible states. Under such conditions, realised trajectories through occupancy space are naturally path-dependent.

**S5.4 Local deformation and curvature**

Because the occupancy field is defined over a structured state space, geometric quantities may be introduced on it. Curvature may be used as a mathematical metric for describing local deformation of realised redox state.

Let $\rho(x,t)$ denote the local occupancy configuration. A change in local realised redox state corresponds to a deformation of this configuration over the underlying abstract state space. Curvature is introduced here as a mathematical device for characterising such deformation. It is not a physical force and does not imply a specific biochemical mechanism.

In this framework, curvature provides a compact way to describe how realised occupancy departs from a locally flat or reference configuration. Regions of high deformation may correspond to sharp local redistribution of realised state, whereas flatter regions may correspond to more stable or weakly varying occupancy structure.

**S5.5 Persistence, memory, and decay**

The geometric formalism also provides a natural basis for representing persistence and memory. If local deformation of the occupancy field does not relax instantaneously, then past realised flux may continue to influence present local redox state. In this sense, curvature may encode biochemical memory as a retained deformation of realised occupancy.

A simple way to formalise this is to introduce a decay or relaxation timescale $\tau$for local deformation. Schematically, one may write

$$\partial_{t}\rho(x,t\mathcal{)=F(}\rho,w,x,t)-\frac{1}{\tau}\text{ }\mathcal{D[}\rho(x,t)],$$

where $\mathcal{F}$ denotes flux-induced evolution and $\mathcal{D}$ a deformation or relaxation term. The precise form of $\mathcal{D}$is left representation-dependent, since different biochemical systems may exhibit different mechanisms of persistence or relaxation.

This provides a formal route by which known processes such as protein turnover, protein degradation, irreversible modification, or slow resetting of redox-sensitive states may be represented as decay of local occupancy deformation rather than as separate ad hoc additions to the theory.

**S5.6 Attractors and path-dependent behaviour**

Because the occupancy field evolves over time and may retain deformation, the geometry layer provides a natural substrate for attractors and related dynamical structures. Stable realised configurations of $\rho(x,t)$ may be interpreted as attractor-like regions in occupancy space, while transitions between them may depend on the trajectory by which the system arrived there.

Path dependence therefore arises naturally in the geometry layer. Two systems with identical present control conditions may nevertheless occupy different local redox configurations if they arrived there through different realised flux histories. Hysteresis is one important consequence of this possibility.

**S5.7 Spatial coupling**

Spatial coupling may be introduced through admissible transport processes that redistribute local realised occupancy across the domain. Examples include diffusion, directed transport, compartmental exchange, or other locality-preserving transport processes. These processes do not alter the admissible structural primitive itself; they redistribute realised occupancy over space.

Hence, geometry enters the theory by localising realised redox state, while transport governs how local occupancy patterns couple across neighbouring regions.

**S5.8 Summary**

The geometry layer represents realised redox state as a bounded informational occupancy field over space and time. This construction introduces locality without postulating a new physical field. Because the field is bounded and structured, it naturally admits piecewise-linear and tropical interpretations. Because it is geometric, curvature may be introduced as a mathematical device for describing deformation, persistence, memory, attractors, and related path-dependent behaviour.
